# Discontinuity in Equilibrium Wave‐Current Ripple Size and Shape and Deep Cleaning Associated With Cohesive Sand‐Clay Beds

**DOI:** 10.1029/2022JF006771

**Published:** 2022-09-23

**Authors:** X. Wu, R. Fernandez, J. H. Baas, J. Malarkey, Dan. R. Parsons

**Affiliations:** ^1^ Energy and Environment Institute University of Hull Hull UK; ^2^ Department of Civil and Environmental Engineering The Pennsylvania State University State College PA USA; ^3^ School of Ocean Sciences Bangor University Menai Bridge UK; ^4^ Loughborough University Leicestershire UK

## Abstract

Mixtures of cohesive clay and noncohesive sand are widespread in many aquatic environments. Ripple dynamics in sand‐clay mixtures have been studied under current‐alone and wave‐alone conditions but not combined wave‐current conditions, despite their prevalence in estuaries and the coastal zone. The present flume experiments examine the effect of initial clay content, *C*_0_, on ripples by considering a single wave‐current condition and, for the first time, quantify how changing clay content of substrate impacts ripple dimensions during development. The results show inverse relationships between *C*_0_ and ripple growth rates and clay winnowing transport rates out of the bed, which reduce as the ripples develop toward equilibrium. For *C*_0_ ≤ 10.6%, higher winnowing rates lead to clay loss, and thus the presence of clean sand, far below the base of equilibrium ripples. This hitherto unquantified “deep‐cleaning” of clay does not occur for *C*_0_ > 10.6%, where clay‐loss rates are much lower. The clay‐loss behavior is associated with two distinct types of equilibrium combined flow ripples: (a) Large asymmetric ripples with dimensions and plan geometries comparable to their clean‐sand counterparts for *C*_0_ ≤ 10.6% and (b) small, flat ripples for *C*_0_ > 10.6%. The 10.6% threshold, which may be specific to the experimental conditions, corresponds to a more general 8% threshold found beneath the ripple base, suggesting that clay content here must be <8% for clean‐sand‐like ripples to develop in sand‐clay beds. This ripple‐type discontinuity comprises a threefold reduction in ripple height, with notable implications for bed roughness.

## Key Points

- Ripple growth rates decrease with increasing initial bed clay content in sand‐clay substrates under combined, wave‐current, flows
- Small, flat ripples occur when the clay content is above an 8% threshold below the ripple base, as strong cohesion prevents ripple growth
- Large clean‐sand‐like ripples form below the clay threshold, with deep winnowing (cleaning) of clay and large equivalent clean‐sand depths

## 1 Introduction

Ripples are primary sedimentary structures that are ubiquitous on the bed of estuaries and coastal seas. These bedforms often preserve information of the flow parameters by which they were formed (e.g., Soulsby & Clarke, 2005; Southard, 1991). Ripple‐related bed roughness in turn modifies near‐bed hydrodynamics and turbulence, ultimately affecting sediment fluxes, a process which is essential for the modeling of sediment transport (e.g., Soulsby, 1997; Van Rijn, 2007). Many estuarine and coastal environments face extreme weather events, which are predicted to increase in frequency with rising sea levels (e.g., Woodruff et al., 2013). Storm‐induced waves combined with currents cause particularly dynamic ripple behavior and thus large and rapidly changing sediment transport rates (e.g., Li & Amos, 1999; Wengrove et al., 2018). The understanding of how hydrodynamics control ripple dimensions is therefore essential for ensuring the improved performance of coastal morphodynamic models through well‐parameterized bed roughness and for process‐scale models of ripple development and stability (Jin et al., 2022; Marieu et al., 2008). This may also be beneficial for the improvement of estuarine and coastal management within the broader context of climate change and sea level rise in coastal systems. Furthermore, using inverse relationships to predict hydrodynamic variables from ripples preserved in sedimentary rocks is essential for the accurate reconstruction of paleoenvironments (e.g., Myrow et al., 2018; Rubin & Carter, 2005). Finally, in view of the nutrient cycle of the coastal ecosystem, ripples have a significant effect on the exchange of dissolved substances between the water column and the seabed, providing organic matter to benthic communities and returning the decompositional products as nitrogen resources for phytoplankton (e.g., Huettel et al., 1996; Nedwell et al., 1993; Snelgrove & Butman, 1995).

Flume studies have provided high‐quality process information concerning ripple dynamics on beds composed of well‐sorted clean sand under steady currents (e.g., Baas, 1994, 1999), waves (e.g., Pedocchi & García, 2009; O'Hara Murray et al., 2011) and combined wave‐current flows (e.g., Dumas et al., 2005; Perillo, Best, Yokokawa, et al., 2014). Empirical formulae developed for the prediction of ripple size have been derived from clean‐sand ripples in laboratories and at field sites (e.g., Khelifa & Ouellet, 2000; Lapotre et al., 2017; Nelson et al., 2013). However, these ripple size predictors are of limited use for the majority of estuarine and coastal environments, where sediment almost universally consists of mixtures of cohesive clay and noncohesive sand (Healy et al., 2002). Recently, researchers have therefore focused on ripple dynamics within substrates composed of mixtures of sand and clay. For steady currents with a depth‐averaged velocity of c. 0.36 m/s, Baas et al. (2013) found that equilibrium ripple height decreased with increasing initial clay content. Wu et al. (2018) highlighted that a small increase in clay content, from 4.2% to 7.4%, exponentially increased the time needed for ripples to reach equilibrium under waves with a maximum free stream velocity of c. 0.35 m/s. However, their equilibrium dimensions were independent of the initial clay content in the bed, up to around 8%. Additionally, clay winnowing, a hydrodynamic sorting process which suspends the finer clay but leaves the coarser sand in the bed (e.g., Cizeau et al., 1999), played a significant role in the transformation of ripples in mixed sand‐clay to an increasingly sandy composition in the experiments of Baas et al. (2013) and Wu et al. (2018). Importantly, Baas et al. (2019) have recently highlighted the role of bed cohesion in decreasing current ripple dimensions in the Dee Estuary, UK, demonstrating that previous laboratory findings are applicable in a qualitative sense in natural environments. There has, however, been very little research on the dynamics of ripples in mixed sand‐clay beds under combined wave‐current flows, which are crucial to the sediment dynamics in the majority of estuaries and coastal seas. This paper therefore extends the experimental work of Baas et al. (2013) and Wu et al. (2018) by providing the first results of the influence of cohesive clay on ripple dynamics by means of flume experiments using a single, common, wave‐current condition. The three specific objectives were: (a) To quantify ripple development for different initial bed clay fractions; (b) to determine the relationship between the equilibrium ripple dimensions and initial bed clay content; and (c) to relate the ripple development to the changing bed composition, based on quantifying, for the first time, clay winnowing from the bed into the water column.

## 2 Materials and Methods

### 2.1 Experiment Setup

**Figure 1:**
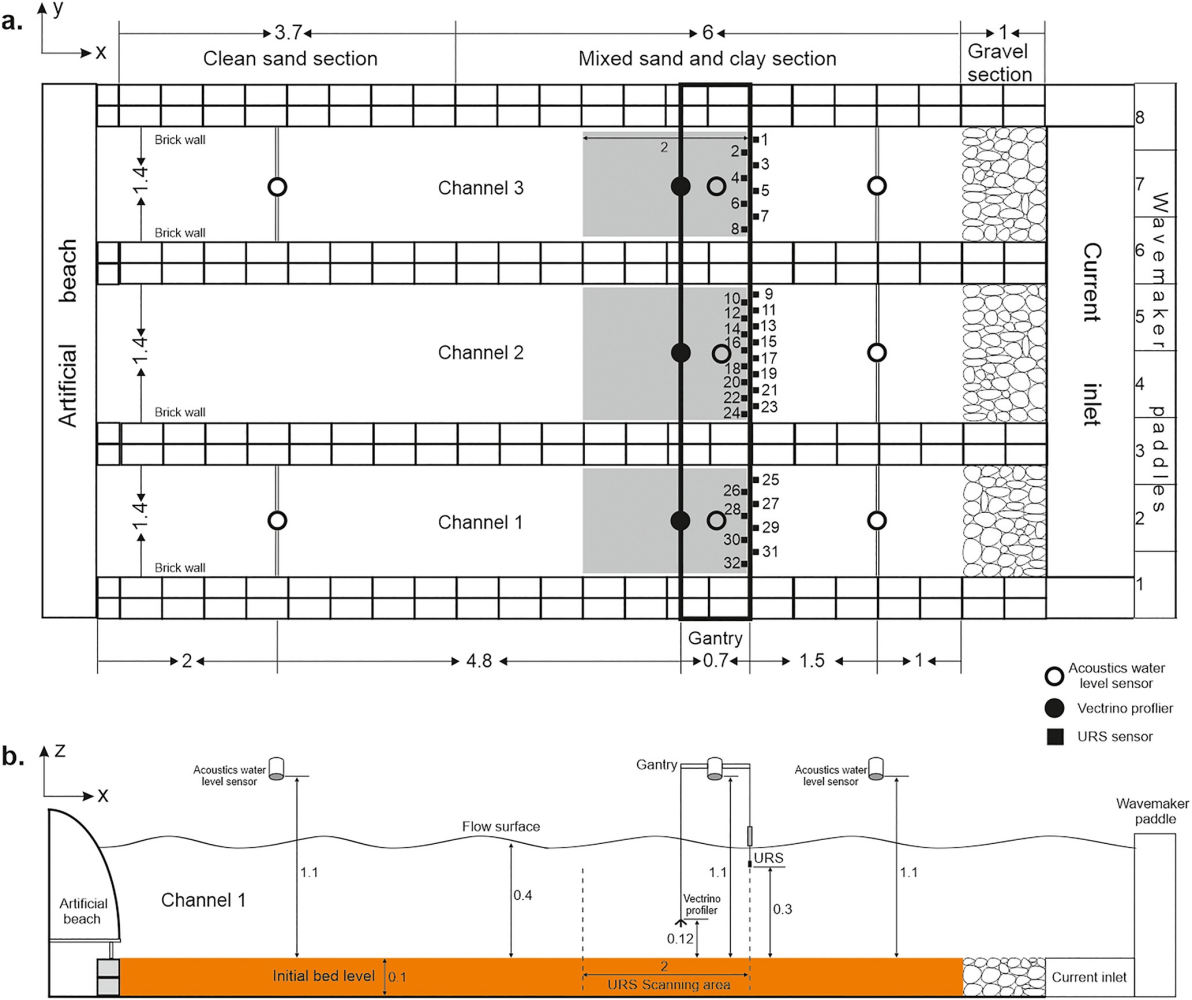
(a) Plan view and (b) side view of the experimental setup. The gray area is scanned by an Ultrasonic Range System (URS) with numbered sensors (black squares). White and black circles denote acoustic water level sensors and Vectrino profilers, respectively. Dimensions are in meters.

A series of large flume experiments were conducted in the Total Environment Simulator at the University of Hull. Three channels of equal size (11 m in length and 1.4 m in width, with brick walls 0.2 m in height) were constructed in a recirculating tank to allow experiments with three distinct clay fractions to be run simultaneously at the same flow conditions. The tank had a 1‐m long gravel section at the upstream end to allow for boundary layer development and an artificial beach made of polyethylene foam at the downstream end to damp out wave reflections (Figure 1a). A cross‐tank mobile gantry centered 3.5 m downstream of the inlet and capable of traversing 2 m downstream held most of the measurement sensors. A flat sediment bed, 0.1 m thick, was present in each channel at the start of the experiments. Fresh water was used in all experiments, and the water depth, *h*, was set to 0.4 m in the test section. Control Run one used three beds of well‐sorted sand with a median diameter, *D*_50_, of 450 μm. Runs two and three, which considered the effect of physical cohesion, used a homogenous mix of kaolinite clay with *D*_50_ = 8.9 μm and the same sand. Six beds were prepared with initial clay content ranging from 5.7% to 12.3% by dry weight (Table 1).

**Table 1:** Experimental Parameters

Run	Duration (min)	Channel^a^	*C*_0_ (%)	*U*_*o*_ (m/s)	*U*_*c*_ (m/s)	*U*_max_ (m/s)	*U*_min_ (m/s)
1	1,970	2	0	0.32	0.16	0.45	−0.01
2	2,000	2	10.6	0.31	0.15	0.44	−0.01
		3	12.3	0.33	0.20	0.51	−0.01
3	1,250	1	5.7	0.32	0.19	0.4	−0.08
		2	8.5	0.31	0.16	0.45	−0.01
		3	11.6	0.33	0.19	0.50	−0.01

^a^ Data for channel one of Run two is excluded because clay and sand were not mixed homogeneously.

Each run was carried out using combined flows. Flow velocities in each channel were measured by a 25 Hz Vectrino profiler fixed on the gantry beam 4.5 m away from the flume inlet and at 0.12 m above the initial flatbed (Figure 1a). The monochromatic wave height, *H*, and wave period, *T* were c. 0.17 m and 2 s, respectively, measured by eight 50 Hz acoustic water level sensors 0.7 m above the still water surface in the tank (Figure 1b). The wave velocity amplitude at the edge of the boundary layer, *U*_o_, was c. 0.32 m/s, and the depth‐averaged current velocity, *U*_c_, was c. 0.16 m/s. Although there was a small variation in velocities across the flume tank (Table 1), this did not significantly influence ripple evolution. The waves in the experiments were skewed shallow water waves with sharp crests and long troughs, generating a maximum combined flow velocity, *U*_max_, of c. 0.45 m/s and a minimum combined flow velocity, *U*_min_, of c. −0.01 m/s at a height above the bed of 0.05 m, when combined with the current (Table 1). Closer to the bed, using the approach of Malarkey and Davies' (2012) strongly nonlinear option for calculating the mean bed shear stress, *τ*_m_, and maximum bed shear stress, *τ*_max_, associated with a skin friction roughness of 2.5*D*_50_ (*z*_0_ = 2.5*D*_50_/30, where *z*_0_ is the theoretical height of zero velocity) in wave‐current flow determined from *h*, *U*_*o*_, *T*, and *U*_*c*_ gives *τ*_max_ = 1.41 Pa and *τ*_m_ = 0.14 Pa. The minimum bed shear stress, *τ*_min_ = *τ*_m_–*τ*_max_, was −1.27 Pa, indicating that bed shear stress was far more symmetric in the two wave half cycles than the velocity higher up in the flow.

The nondimensional Shields parameter is defined by *θ* = *τ*/(*ρ*_*s*_–*ρ*)*gD*_50_, where *ρ*_*s*_ is the sediment density (=2650 kg/m^3^), *ρ* is the water density (=1,000 kg/m^3^ for freshwater and 1,027 kg/m^3^ for saltwater), and *g* is the acceleration due to gravity (=9.81 m/s^2^). During the intertidal Dee Estuary (UK) field deployment of Lichtman et al. (2018) and Baas et al. (2021), *D*_50_ = 0.227 mm, 0 < *h* ≤ 3.5 m, 0 < *U*_*c*_ ≤ 0.6 ms^−1^, 0 < *U*_*o*_ ≤ 0.45 ms^−1^, and 0 < *C*_0_ ≤ 14%. During a storm event (tidal inundations two to six of Lichtman et al. (2018) and Baas et al. (2021)), the peak *τ*_max_, calculated in the same way as above, varied between 0.55 and 1.69 Pa and the clean‐sand Shields parameter threshold, *θ*_0_, according to Soulsby (1997) was 0.05 or 0.15 ≤ *θ*_max_ ≤ 0.47 and 3 ≤ θ_max_/*θ*_0_ ≤ 9. For the present experiments, the values of *h*, *U*_*c*_, *U*_*o*_, and *C*_0_ were all within these ranges (Table 1), and since *θ*_max_ = 0.19, *θ*_0_ = 0.03, and *θ*_max_/*θ*_0_ ≈ 7, the shear stress was also within the field range. Thus the experimental conditions herein are consistent with intertidal storm conditions in a macrotidal estuary.

### 2.2 Procedure

The clay was homogeneously mixed into the sand using a handheld plasterer's mixer in each channel, flattened using a wooden leveler and scanned using a terrestrial 3D laser scanner (FARO Focus3D X330). At the start of each experiment, syringe‐type sediment cores with a diameter of 20 mm and a maximum length of 90 mm were collected from six locations at 1‐m intervals along the center lines of the mixed sand‐clay sections. A homogenous sand‐clay mix was present in all channels (Figure S1), except for one substrate in Run two, Channel one, which was therefore excluded from the results presented below.

Bedform evolution was recorded in three dimensions using a Seatek 2 MHz Ultrasonic Ranging System (URS) mounted on the gantry 4 m downstream of the flume inlet and 0.3 m above the bed. The URS contained 32 probes spatially distributed across the three channels (16 across Channel two and eight across Channels one and three). During the experiment, the URS probes were static and positioned 3.5 m downstream from the current inlet to monitor ripple migration rate, *m* = *n* *λ*/*t*, where *n* is the number of ripples migrating below the URS probes over a time period *t* (*t* = 60 min). Additionally, every time the flow was temporarily stopped, the array scanned the bed over a 2‐m length swathe via an auto‐traverse system with a speed of 1 mm/s (Figure 1).

Bed scanning was conducted at preset time intervals, gradually increasing from a 5‐min interval in the initial phases of the runs up to 180 min in the later phases of the runs. Sediment syringe cores from the mixed clay and sand sections were also collected during the experiments while the waves and currents were temporarily stopped. In Run two, sediment cores were collected at two locations within the 2‐m scan swathe: Near the start and near the end. One more collection location from the middle of the swathe was added in Run three because ripples with lower clay content were expected to develop faster and an additional sampling point was deemed beneficial to quantifying winnowing. At each collection location, one core was taken from the initial flatbed and as soon as ripples were identified with the URS, a core was collected from a crest and a consecutive trough. After each experiment, the water was drained slowly from the tank and the rippled bed was scanned with the 3D laser scanner, and sediment cores from neighboring ripple crests and troughs were also collected. Additional sediment cores were collected from the ripples in the downstream clean‐sand section in order to quantify the amount of clay that had reentered the sandy substrate by hyporheic processes. All sediment cores were stored in a cold store at a temperature of 4°C prior to grain size analysis using a Malvern Mastersizer 2000. The sediment cores from the initial flatbed and from the ripple troughs were sliced in 10‐mm intervals for the grain size analysis; the cores obtained from the ripple crests were sliced in 5‐mm intervals to provide higher resolution of the clay content within the ripples.

### 2.3 Postprocessing of Data

Ripple wavelengths, *λ*, and heights, *η*, were determined from the bed elevation profiles (BEP), recorded by each URS sensor. The removal of spikes from the raw BEPs was based on d*z* > d*z*_*m*_, where d*z* is the vertical distance between consecutive data points in the BEP and d*z*_*m*_ is the average vertical distance in the BEP (Van der Mark et al., 2008). Each BEP was then smoothed using a three‐point moving average, followed by applying MATLAB^®^ “peaks and troughs” tool to identify the locations of ripple crests and troughs. The end‐of‐experiment cross‐sectional shape of the ripples was characterized by calculating the ripple steepness (RS) and ripple symmetry index (RSI):

(1) RS=η/λ,

(2) $$RSI=\lambda_{s}/\lambda_{l},$$

where *λ*_*s*_ and *λ*_*l*_ are the lengths of the stoss side and lee side, respectively. RSI values between 1 and 1.3 denote symmetric ripples and RSI values higher than 1.5 denote increasingly asymmetric ripples. Ripples are quasi asymmetric for 1.3 < RSI < 1.5 (Perillo, Best, & Garcia, 2014).

Furthermore, the mean values of *η*_*t*_ and *λ*_*t*_ at a bed scanning time *t* were calculated from all ripples in the BEPs in each channel, in order to construct development curves of ripple height and wavelength. Equilibrium ripple height, *η*_*e*_, and wavelength, *λ*_*e*_, and the time required to reach equilibrium height, *T*_*η*_, and wavelength, *T*_*λ*_, were calculated using best‐fit equations proposed by Baas et al. (2013), which include a delay time for the first appearance of ripples on the flatbed, *t*_*i*_:

(3) $$\frac{\eta_{t}-\eta_{i}}{\eta_{e}-\eta_{i}}=1-0.1^{\frac{t-t_{i}}{T_{\eta }-t_{i}}},$$

(4) $$\frac{\lambda_{t}-\lambda_{i}}{\lambda_{e}-\lambda_{i}}=1-0.1^{\frac{t-t_{i}}{T_{\lambda }-t_{i}}},$$

where *η*_*e*_, *λ*_*e*_, *T*_*η*_, *T*_*λ*_, *λ*_i_, and *t*_*i*_ are fitting coefficients, and *λ*_i_ is the initial wavelength of the first ripples that appeared on the flatbed in each run. The initial ripple height, *η*_*i*_, is zero except when more than one growth stage is fitted to the data. In this study, the equilibrium time was defined as the time taken for the ripple wavelength or height to reach 90% of its equilibrium value (cf., Baas et al., 2013). The coefficient *t*_*i*_ was zero in the control run with clean sand (Table 2). All the fitting coefficients for the combined‐flow ripples are listed in Table 2 and discussed in Section 3. The characteristic ripple height growth rate, *r*_*η*_, and wavelength growth rate, *r*_*λ*_, over the experiment were estimated as follows:

(5) $$r_{\eta }=\eta_{e}/T_{\eta },$$

(6) $$r_{\lambda }=\left(\lambda_{e}-\lambda_{i}\right)/T_{\lambda }.$$

**Table 2:** Ripple Parameters

Run/Channel	*C*_0_ (%)	*η*_*e*_ (mm)	*T*_*η*_ (min)	*r* ^2^ (−)	*λ*_*e*_ (mm)	*λ*_*i*_ (mm)	*T*_*λ*_ (min)	*r* ^2^ (−)	*t*_*i*_ (min)	RSI (−)	RS (−)
1/2^a^	0	14.4 ± 1.8	90	0.78	123.6 ± 4.9	80.7 ± 10.8	170	0.94	‐	1.4 ± 0.3	0.12 ± 0.02
3/1	5.7	14.7 ± 1.1	125	0.74	126.5 ± 3.5	91.3 ± 6.3	330	0.88	5	1.4 ± 0.3	0.12 ± 0.02
3/2	8.5	14.3 ± 1.0	432	0.96	121.4 ± 5.1	80.2 ± 11.6	456	0.85	60	1.4 ± 0.3	0.11 ± 0.02
2/2	10.6	13.7 ± 1.0^b^	678^b^	0.92	110.9 ± 4.0^b^	92.6 ± 10.2^b^	540^b^	0.63	60^b^	1.3 ± 0.3	0.14 ± 0.03
3/3	11.6	4.1 ± 0.4	271	0.76	108.5 ± 2.6	84.5 ± 10.	382	0.78	90	1.5 ± 0.5	0.05 ± 0.02
2/3	12.3	3.5 ± 0.3	211	0.79	98.0 ± 1.7	71.7 ± 6.3	499	0.93	120	1.5 ± 0.5	0.04 ± 0.02

^a^ The first and second number represents run and channel number, respectively, for example, 1/2 for Run 1 Channel 2.

^b^ Based on a two‐stage fitting (first stage for *t* ≤ 230 min, with *η*_*i*_ = 0 mm and *λ*_*i*_ = 46 mm and second stage for *t* > 230 min, with *η*_*i*_ = 3.8 mm and *λ*_*i*_ = 92.6 mm, the quoted value of *T*_*η*_ and *T*_*λ*_ includes the time of the first stage, 230 min).

*Note*. *r* ^2^: Squared correlation coefficient of the best‐fit curve; ±: Standard deviation.

Assuming that these rates are small compared to the ripple migration rate, *m*, which will be demonstrated later, the rate of removal of clay out of the bed does not have to take changes in ripple dimensions into account. The total amount of clay removed since the beginning of the experiment, *I*, can be estimated by the following equation:

(7) $$I=\int_{-b}^{0}wC_{def}dz,$$

where *z* = 0 and *z* = –*b* correspond to the ripple crest and the lowest reference levels of the sediment cores, respectively, *C*_def_(*z*) is the clay deficit in the bed, compared to *C*_0_, given by *C*_def_ = *C*_0_–*C*, and *C* is the measured clay content in the sediment cores, such that *I* = 0 for the initial core by definition. The weighting function *w*(*z*) = –*z*/*η*, for the active layer (–*η* < *z* < 0), and *w*(*z*) = 1 for *z* ≤ −*η* and *z* = −*η* corresponds to the ripple trough depth (the vertical offset for the trough core). The weighting function represents the fraction of the bed taken up by the ripple, assuming it has a triangular cross section. Equation 7 also allows for the definition of an equivalent clean‐sand depth *d*_*c*_ = *I*/*C*_0_, which is the effective depth to which clay has been removed. This quantity can be compared to the ripple height.

The mass transport rate of clay per unit width out of the bed, *T*_*b*_, was determined by the following equation:

(8) $$T_{b}=\left(1-p\right)\rho_{s}\lambda \frac{∆I}{∆t}\;$$

where *p* = 0.4 is the closest packing porosity, and ∆*I* and ∆*t* are the changes in *I* and time, *t*, between sequential cores.

Finally, in order to characterize its behavior, the clay concentration in the bed is fitted to a Gaussian type function:

(9) $$C\left(z\right)=\left\{\begin{array}{ll} C_{s}, & -z_{s}\leq z<0,\\ C_{0}-\left(C_{0}-C_{s}\right)\exp\left[-\alpha {\left(z+z_{s}\right)}^{2}\right], & -b\leq z<-z_{s}, \end{array}\right.$$

where *C*_*s*_ is the clay concentration at the surface, *z*_*s*_ is the height above which the clay concentration is constant (*z*_*s*_ ≤ *η*), *α* is the decay constant, and *b* is set to a fixed depth of 100 mm for all profiles.

## 3 Results

### 3.1 Ripple Development

**Figure 2:**
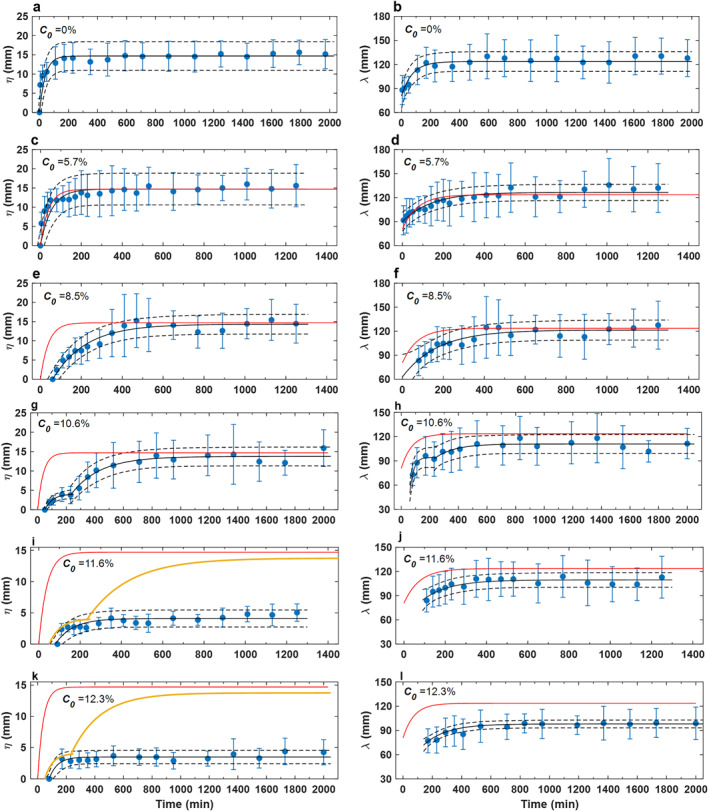
Development trends for (a, c, e, g, i, and k) ripple height and (b, d, f, h, j, and l) ripple wavelength. Blue vertical lines denote one standard deviation of the mean dimension. Black lines are based on fitting to Equations 3 and 4. Red and yellow lines are best‐fit curves for clean sand and 10.6% cases, for comparison. Black dash lines represent the 95% confidence interval of the fitted functions. Note that the 10.6% case involves a two‐stage fitting (the first for *t* ≤ 230 min with *η*_*i*_ = 0 and *λ*_*i*_ = 46 mm and the second for *t* > 230 min with *η*_*i*_ = 3.8 and *λ*_*i*_ = 92.6 mm, see Table 2).

**Figure 3:**
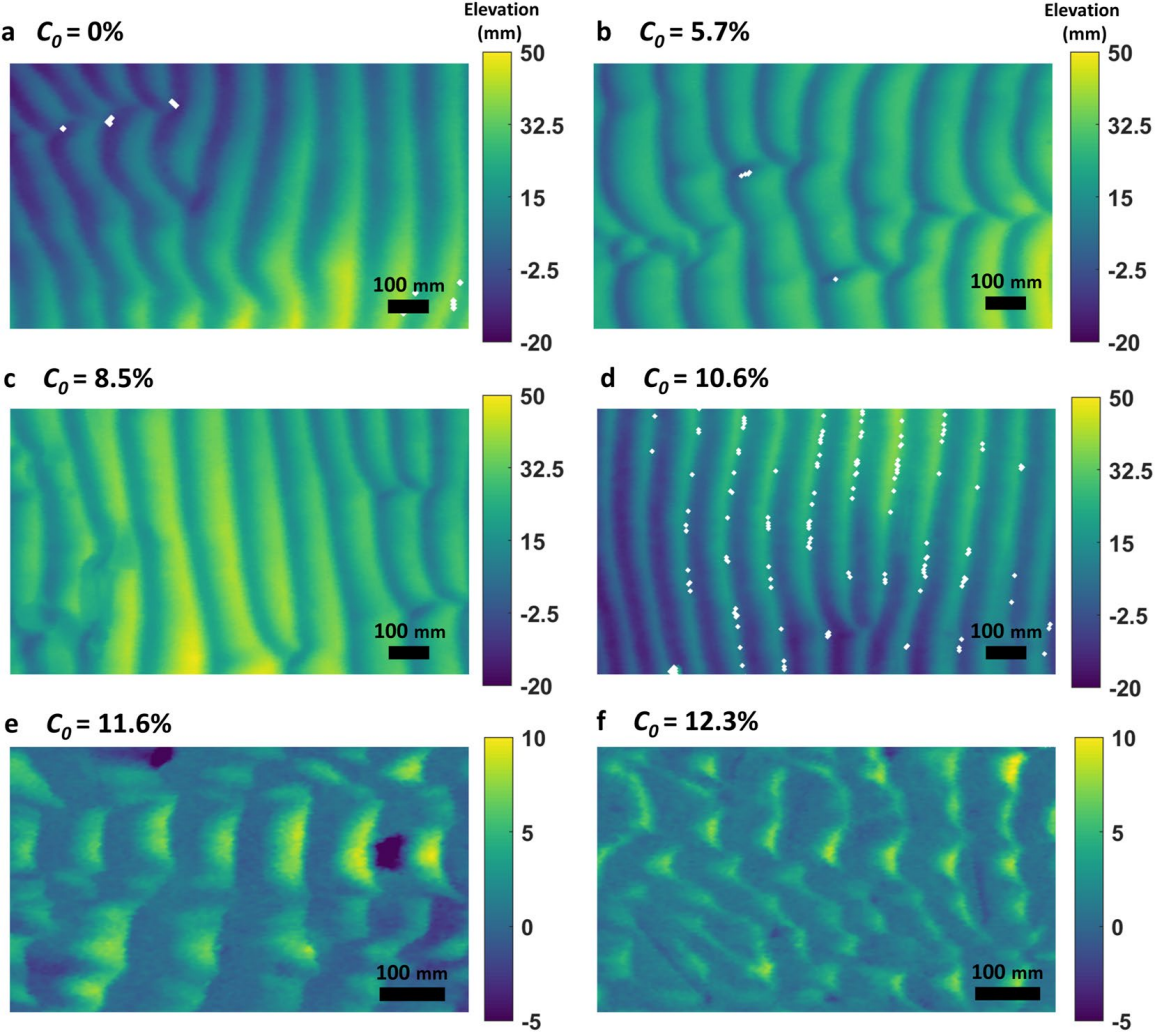
Plan view of the ripple morphology at the end of the experiments in the test section. *C*_0_ is the initial bed clay content.

During the control run (*C*_0_ = 0%), small ripples appeared on the flatbed immediately after the hydrodynamic forcing was applied, as evidenced by a 5‐min period of rapid growth, during which the ripple wavelength and height reached 88.1 and 7.2 mm, respectively. Thereafter, the ripple growth rate progressively declined until the ripples stabilized (Figures 2a and 2b). The development of these ripples exhibited a general trend similar to that reported in the combined‐flow experiments of Perillo, Best, Yokokawa, et al. (2014). Equations 3 and 4 revealed that the ripples took 90 and 170 min to reach an equilibrium height and wavelength of 14.6 and 123.6 mm, respectively, with high confidence fits of 0.94 and 0.78 (Figures 2a and 2b; Table 2). These fully developed ripples were two‐dimensional in planform geometry, characterized by straight, continuous ripple crest lines (Figure 3a). The majority of the ripples were symmetric or quasi asymmetric, with a RSI of 1.4 and a RS of 0.12 (Table 2), indicating that they were similar to wave‐generated vortex ripples (Miller & Komar, 1980).

The ripples for the lowest bed clay content, *C*_0_ = 5.7% (Run three, Channel one), had a similar growth rate to that of the clean‐sand ripples in the first 50 min (Figures 2c and 2d). Thereafter, the ripple growth rate reduced compared to the counterpart in clean sand, such that it took longer to reach equilibrium: 125 min for the ripple height and 330 min for the ripple wavelength. The equilibrium dimensions, *η*_*e*_ = 14.7 mm and *λ*_*e*_ = 126.5 mm, were similar to the clean‐sand ripples, as was their morphology, with two‐dimensional ripples covering the bed (Figure 3b).

As *C*_0_ was increased, *t*_*i*_ increased from 60 to 120 min (Figure 2e–2l; Table 2). Increasing *C*_0_ also slowed the subsequent ripple development compared with the clean‐sand case (Figures 2e–2l). This is particularly true for *C*_0_ = 10.6%, where the ripple height and wavelength only grew to 3.9 and 96.2 mm at *t* = 170 min, compared to 14.1 and 122 mm for the clean‐sand ripples at the same point in time. In the following hour these small ripples tended to be stable; Equations 3 and 4 also captured the growth trends well in height and wavelength in this stage (Figures 2g and 2h). After *t* = 230 min, the ripple height experienced a period of relatively rapid, yet gradually decelerating, growth in the next approximately 7 hours, reaching *η*_*t*_ = 11.5 mm at *t* = 530 min and *η*_*e*_ = 13.7 mm at *T*_*η*_ = 678 min (Figure 2g; Table 2), which was similar to the equilibrium height of the clean‐sand ripples. Ripple wavelength reached equilibrium *λ*_*e*_ = 110.9 mm at *T*_*λ*_ = 540 min (Figure 2h; Table 2). Not only the dimensions of the ripples were similar to the clean‐sand ripples for *C*_0_ ≤ 10.6%, but these ripples were also two‐dimensional, tended to be slightly asymmetric with RSI ≈1.3, and they had RS = 0.11–0.14 (Figures 3c and 3d; Table 2).

The initial ripple height growth trends for the 11.6% and 12.3% cases were like their 10.6% counterparts until *t* = 230 min (Figures 2i and 2k). However, thereafter, the ripples experienced weak growth in the remainder of the experiments and were unable to develop to sizes similar to the clean‐sand ripples, reaching *η*_*e*_ = 4.1 and *η*_*e*_ = 3.5 mm at *T*_*η*_ = 271 and *T*_*η*_ = 211 min (*r* ^2^ = 0.76 and 0.79), respectively (Figures 2i and 2k; Table 2). Wavelength development was also hindered in these high clay content cases. For the 11.6% case, 280 min were required to reach *λ*_*e*_ = 108.5 mm, whereas a longer period of 499 min was needed to reach a shorter *λ*_*e*_ = 98 mm for the 12.3% case. These differences in ripple dynamics, compared to the clean‐sand ripples, were also reflected in their geometry. For *C*_0_ = 11.6%, the ripples were quasi‐2D, characterized by straight but discontinuous crest lines, whereas barchan‐shaped ripples with discontinuous crest lines were observed for *C*_0_ = 12.3% (Figures 3e and 3f). Both these ripple types were more asymmetric, with RSI = 1.5, and markedly flatter, with RS ≈0.05, than the clean‐sand ripples (Table 2).

**Figure 4:**
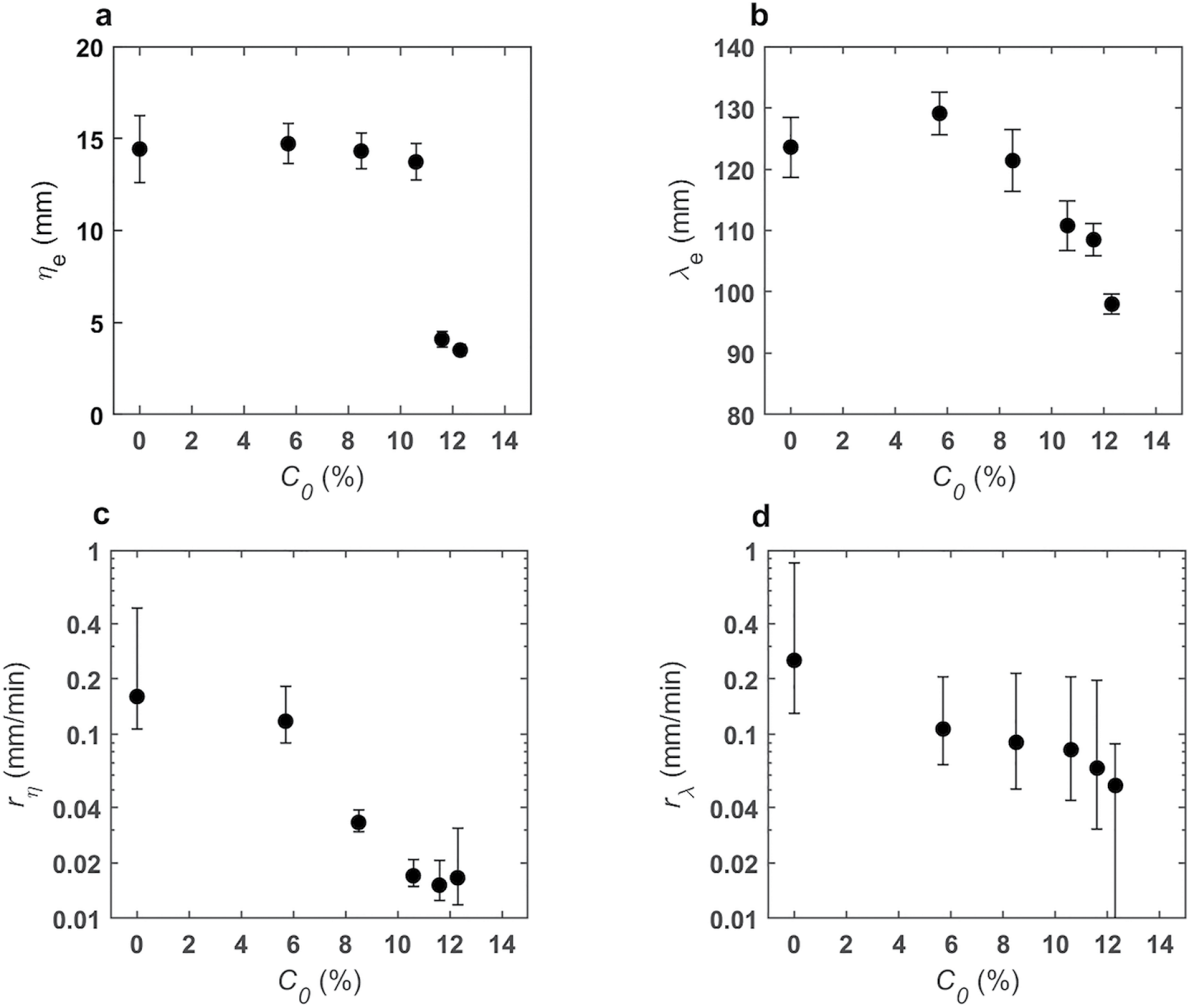
Equilibrium combined‐flow (a) ripple height and (b) wavelength and development rate of (c) ripple height and (d) wavelength against initial bed clay content for all experiments. Black bars denote 95% confidence intervals, derived from best‐fit Equations 3 and 4.

Figure 4 illustrates the relationship between the initial clay content and the principal properties of the equilibrium combined‐flow ripples. The equilibrium ripple height was almost independent of the initial clay content for *C*_0_ ≤ 10.6%, at *η*_*e*_ ≈ 14.4 mm, whereas *η*_*e*_ collapsed to 3.5 mm at the highest *C*_0_ of 12.3%, almost four times smaller than the clean‐sand equilibrium height (Figure 4a). The equilibrium wavelength was between 121.4 and 129.1 mm for *C*_0_ ≤ 8.5% and declined linearly at higher *C*_0_ values, that is, from 110.8 mm at 10.6% to 98 mm at 12.3% (Figure 4b). The growth rate decreased gradually between 0% and 12.3% clay (Figures 4c and 4d). In the clean‐sand run, *r*_*η*_ and *r*_*λ*_ were 0.16 and 0.25 mm/min, respectively. At *C*_0_ = 12.3%, the growth rates were up to an order of magnitude lower at *r*_*η*_ = 0.017 mm/min and *r*_*λ*_ = 0.052 mm/min. Based on an equilibrium migration rate of 4–9 ripple crests/h (Fernández et al., 2022) and the range of ripple wavelengths of 71–132 mm, this gives a migration rate of *m* = 5–20 mm/min. The migration rate is at least one order of magnitude greater than the ripple height and wavelength growth rates, therefore it is justified to use Equations 7 and 8 for the transport rate of clay sediments out of the bed, as the ripples remained unchanged when moving one ripple wavelength.

### 3.2 Change in Bed Clay Content With Ripple Development

**Figure 5:**
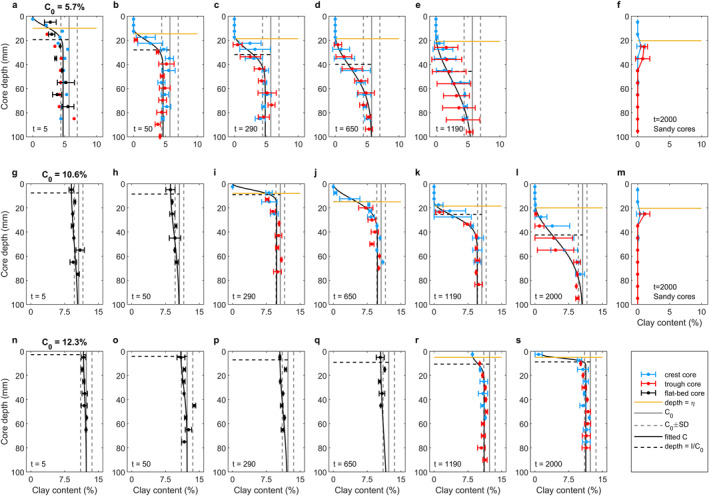
Vertical profiles of clay content in cores collected from beds in the mixed sand‐clay section with clay content of (a–e) 5.7%, (g–l) 10.6%, and (n–s) 12.3% and from end‐of‐experiment rippled beds in the clean‐sand section downstream of the three channels in (m) Run two and (f) Run three. The gray vertical solid lines and the vertical dashed lines represent mean initial clay content and one standard deviation of the mean (See Figure S1). The black, blue, and red dots denote mean clay content below the active flatbed, ripple crest, and ripple trough, respectively. The black, blue, and red horizontal bars denote one standard deviation of the mean clay content. The solid black line is the fit to Equation 9 (for 5.7% and *t* = 5 min, the flatbed core is not used in the fit) and the dashed horizontal black line is *d*_*c*_ = *I*/*C*_0_, the equivalent clean‐sand depth. The yellow lines represent the ripple base and *t* is the time at which the core was taken in min.

Figure 5 shows representative examples of the changes in bed clay content for different stages in the development of the ripples for *C*_0_ = 5.7%, 10.6%, and 12.3% based on the grain‐size analysis of the sediment in the cores. The first profile for *C*_0_ = 5.7% was at *t* = 5 min, when the bed was partly flat and partly occupied by small ripples. In the upper 15 mm of the flatbed core (black dots in Figure 5a), the clay content was about half of its initial value (gray vertical solid line in Figure 5a). Below this layer, the clay content increased with depth to its initial value (Figure 5a). The sand was free of clay below the crest of the small, 8 mm high, ripples, whereas a small amount of clay remained at the base of the ripple (blue dots in Figure 5a). There was clay loss in a 10‐mm thick layer immediately below the ripple trough, with the clay content c. 60% lower than the initial value (red dots in Figure 5a). At *t* = 5 min, the equivalent clean‐sand depth *d*_*c*_ (=*I*/*C*_0_) was approximately 20 mm (black horizontal dash line), which was much larger than the ripple height, suggesting that there was enough clean sand below the ripple trough to allow ripple growth. Below this layer, the clay content had remained close to its initial value (Figure 5a). Figures 5b–5e illustrates the effect of ripple growth on the bed clay content. The clay content in the rippled part of the cores was zero, indicating that the winnowing of clay from the bed kept pace with the growth in ripple height. Furthermore, the thickness of the layer losing clay just below the ripple base progressively expanded downward until it reached the initial value (*C*_0_) at the base of the deposit between 650 and 1,250 min. Consequently, the thickness of *d*_*c*_ continued to increase, reaching 45.8 mm by the end of the experiment (Figure 5e).

Five minutes after the start of the run with *C*_0_ = 10.6%, the clay content in the top 10 mm of the flatbed core was lower than the initial value but above 7.5%; this layer of slightly reduced clay content had expanded downward to c. 30 mm at *t* = 50 min (Figures 5g and 5h). By *t* = 290 min, the ripples contained 0% clay just below the ripple crest but clay was retained at the ripple base. There was a relatively thin layer (≈10 mm thick) showing a c. 30% reduction in clay content underneath the ripples, with *d*_*c*_ ≈ *η* (Figure 5i). Compared to *C*_0_ = 5.7%, the sediment cores demonstrate a slower evolution toward fully developed sandy ripples in conjunction with a slower downward expansion of the layer with reduced clay content underneath the ripples (Figures 5i–5l). By the end of the experiment, the thickness of the layer losing clay below the ripples was comparable with that of the 5.7% case, as *d*_*c*_ increased to 42.5 mm (Figure 5l).

The bed clay content for the 12.3% run was close to its initial value at *t* = 5 min (Figure 5n). At *t* = 50 min (Figure 5o), the upper 10 mm of the bed had lost a small amount of clay; ripples had not formed at this stage. Small ripples were present at *t* = 290 and 650 min but it was not possible to sample through the crests and troughs of these ripples. Bed clay content in the upper 10 mm had continued to decrease at these times (Figures 5p and 5q). Draining the tank at *t* = 1,190 and *t* = 2000 min revealed small, c. 5 mm high, ripples. These ripples had retained 8.5% clay at *t* = 1,190 min but only 0.8% at *t* = 2,000 min. While a thin layer of reduced clay content was present just below the base of the ripples (Figures 5r and 5s), the initial clay content was recovered at far shallower depths than for the 5.7% and 10.6% cases and *d*_*c*_ was close to *η*.

In the rippled‐bed cases for all three initial concentrations (Figures 5b–5e, 5i–5l, 5r and 5s), fitting to a Gaussian‐type function, Equation 9, by optimizing *C*_0_, *C*_*s*_, *z*_*s*_, and *α* each time step (black line) provides a reasonable description of the data (*R* ^2^ ≥ 0.74). At the deepest point *z* = –*b,* all clay contents are consistently within one standard deviation of the initial clay concentration (gray dashed lines).

Figures 5f and 5m shows vertical profiles of clay content collected from ripples in the downstream clean‐sand sections of the three channels in Run two and three at the end of the experiments. Both profiles reveal clay‐free ripples and a layer modestly enriched in clay below the base of the ripples. This layer was c. 20 mm thick for *C*_0_ = 5.7% and c. 10 mm thick for *C*_0_ = 10.6%.

**Figure 6:**
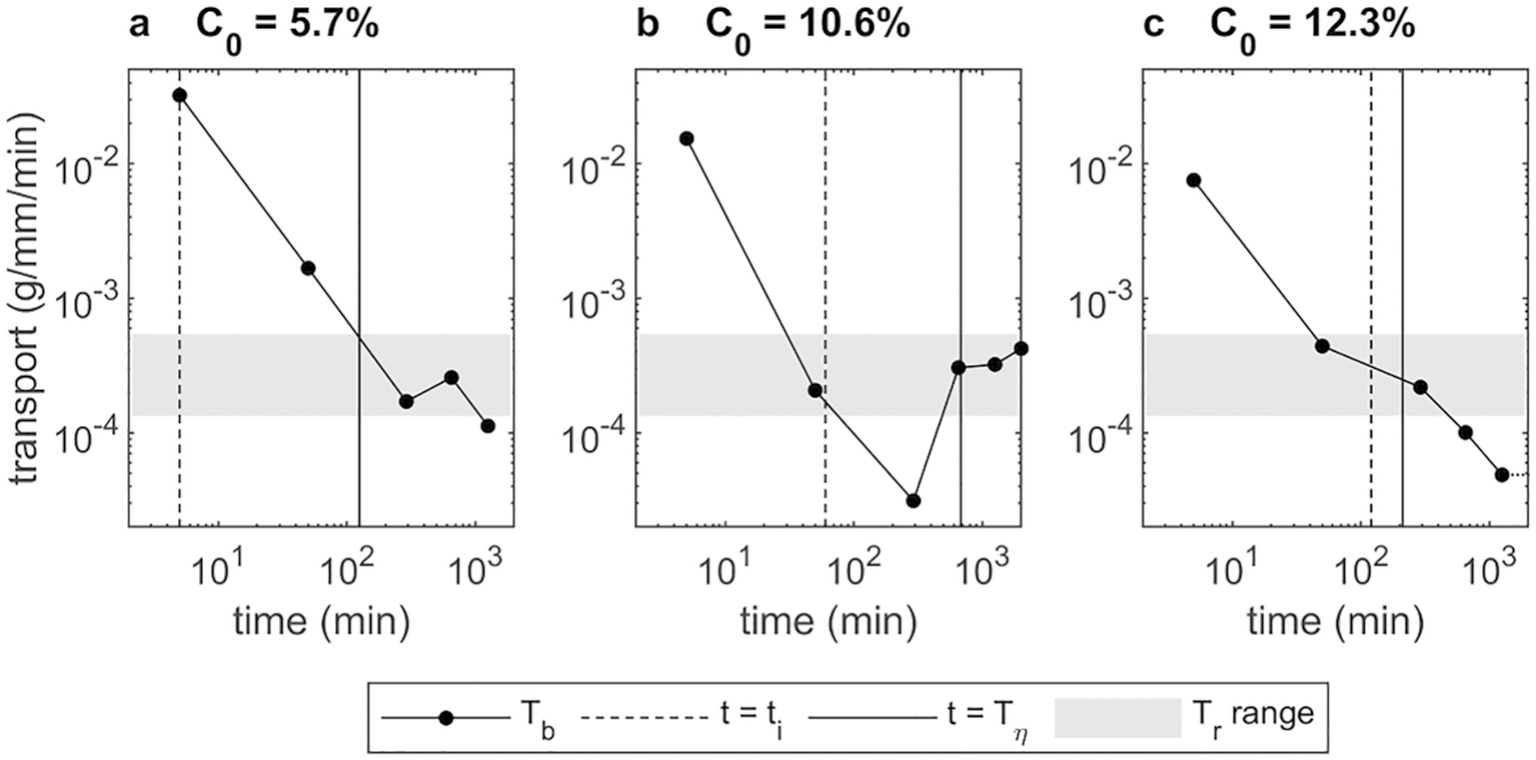
Clay mass transport rate out of the bed, *T*_*b*_, from Equation 8, for *C*_0_ = 5.7% (a), 10.6% (b), and 12.3% (c). Gray shading corresponds to the range of estimated clay mass transport rates in the active layer, *T*_*r*_, due to winnowing induced by ripple migration. Vertical dashed and solid lines correspond to the times of initiation and full development for ripples (*t*_*i*_ and *T*_*η*_) and the dotted line section of *T*_*b*_ for 12.3% indicates where it goes negative.

Figure 6 depicts the clay transport rates out of the bed, *T*_*b*_, which were highest at the beginning of the experiment, where the bed was essentially flat. For *C*_0_ = 5.7% and 10.6%, the transport rates then decreased as the ripples grew and tended to level off after ripples reached equilibrium (Figures 6a and 6b). For *C*_0_ = 12.3%, *T*_*b*_ decreased throughout the experiment, with 4.88 × 10^−5^ g/mm/min at *t* = 1,190 min (Figure 6c). At the end of the experiment *T*_*b*_ even changed sign (dotted line in Figure 6c), indicating that more clay entered the bed than left it through winnowing.

It is anticipated that loss of clay from the rippled beds is largely the result of winnowing from the active layer (−*η* < *z* < 0). For a triangular ripple containing a constant clay concentration, *C*_*r*_, and assuming that all clay present is removed after the passage of one ripple wavelength, this can be expressed as a transport rate, *T*_*r*_ = ½(1−*p*)*ρ*_*s*_ *C*_*r*_ *mη*, where *m* = 5–20 mm/min. Conservatively assuming a modest concentration, *C*_*r*_ = 1%, and the minimum ripple height, *η* = 3.5 mm, gives *T*_*r*_ = 1.3−5.5 × 10^−4^ g/mm/min. Figure 6 shows this range of *T*_*r*_ (gray shading area). It can be seen that *T*_*r*_ was comparable to *T*_*b*_ in the rippled beds, except at *t* = 5 min when the bed was mostly flat, implying that winnowing from the active layer can easily keep pace with the clay being lost from the bed.

The cores collected from the clean‐sand section downstream of the mixed sections have demonstrated that clay can enter the bed through hyporheic processes (Figures 5f and 5m). Using concentrations from Figure 5f and m instead of *C*_def_ in Equation 7 allows a range for hyporheic transport rates over the whole experiment for 5.7% and 10.6% (∆*t* = 1,190 and 2,000 min) of −1.9 and −0.7 × 10^−5^ g/mm/min, that is, into the bed, to be calculated. This is an order of magnitude smaller than the conservative *T*_*r*_ estimate.

## 4 Discussion

### 4.1 Ripple Development on Cohesive Substrates Under Combined Flows

The experimental results described in this paper illustrate the role of cohesive sediment in changing the dynamics of combined‐flow ripples by slowing the ripple growth rate (Figures 4c and 4d; cf. Baas et al., 2013; Wu et al., 2018). For beds with *C*_0_ ≤ 10.6%, the ripples developed to a comparable equilibrium shape and size, with *η*_*e*_ ≈ 14.4 mm and *λ*_*e*_ ≈ 123.8 mm, but the cohesive forces caused the equilibrium time to increase with *C*_0_ for 0% ≤ *C*_0_ ≤ 10.6% (Table 2). These observations are consistent with the findings of Wu et al. (2018), who studied the development of wave ripples on sand beds with up to 7.4% kaolinite clay. However, Baas et al. (2013) described a small decrease in height and a constant wavelength of current ripples, as *C*_0_ was increased from 0% to 12.6% and a similar equilibrium time for all ripples independent of bed kaolinite content. The relatively short duration of 2 hr used in the experiments of Baas et al. (2013) may have prevented the best‐fit equations (cf. Equations 3 and 4) from predicting sufficiently accurate equilibrium times, especially at *C*_0_ values between 7% and 12.6%. This viewpoint is supported by the experiments with mixtures of sand and biologically cohesive extracellular polymeric substances (EPS) of Malarkey et al. (2015), whose flow and sand properties were similar to those of Baas et al. (2013) but their runs lasted between 4 and 73 hr. Malarkey et al. (2015) concluded that current ripples developing on beds with EPS contents ranging from 0.016% to 0.125% reached similar equilibrium size and geometry as EPS‐free current ripples, provided that sufficient time was allowed for their formation. 2D combined‐flow ripples developed in the present experiments, whereas 3D ripples were generated under similar combined‐flow velocities in Perillo, Best, Yokokawa et al. (2014) experiments. This difference in ripple planform geometry is potentially attributable to the grain size used in the experiments; in the present experiment coarser sand, *D*_50_ = 450 μm, was used whereas *D*_50_ = 250 μm was used in Perillo, Best, Yokokawa et al. (2014) experiments. O'Donoghue et al. (2006) found a tendency for 2D ripples to be generated when the grain size exceeded 300 μm. The current component in the combined flow contributed to ripple asymmetry; RSI was around 1.4 in the present experiments, whereas ripples are more symmetric under wave‐alone conditions, for example, RSI ≈ 1.1 in the experiments of Wu et al. (2018). This is also in agreement with previous experimental studies on the influence of combined flow on ripple cross section geometry (Perillo, Best, & Garcia, 2014).

The two strongest levels of bed cohesion (*C*_0_ = 11.6% and 12.3%) used herein not only led to greatly reduced ripple dimensions (*η*_*e*_ < 5 mm, *λ*_*e*_ < 108 mm) but also to significantly different ripple geometries. At RS = 0.04, these small ripples resemble rolling‐grain ripples without flow separation at the crest, as opposed to vortex ripples with flow separation that require RS > 0.1 (Miller & Komar, 1980). Rolling‐grain ripples are associated with steady circulation cells on either side of the ripple crest (Hara & Mei, 1990), which drive sediment toward the crest, causing the ripple to grow until it is steep enough for flow separation and periodic vortex shedding to begin (e.g., van der Werf et al., 2008). Perillo, Best, Yokokawa, et al. (2014) identified small, two‐dimensional rolling‐grain ripples on clean‐sand beds under combined flows. However, the rolling‐grain ripples at *C*_0_ = 12.3% in the present study were barchan‐shaped (Figure 3f). It is likely that the stronger cohesion within the bed at *C*_0_ = 12.3% prevented these ripples from evolving into the two‐dimensional ripples with discontinuous crest lines of *C*_0_ = 11.6% or even the straight‐crested and continuous ripple trains of *C*_0_ ≤ 10.6%. The fact that *T*_*b*_ changes sign in the *C*_0_ = 12.3% case in Figure 6c suggests that the ripples would be unlikely to evolve further, even if the experiment had been run for a longer duration (Dallmann et al., 2021).

Previous experiments have found that clean‐sand rolling‐grain ripples are at a transitional and unstable stage that evolve toward equilibrium vortex ripples (Faraci & Foti, 2001; Scherer et al., 1999; Stegner & Wesfreid, 1999). This transition is usually a rapid process. In the clean‐sand experiments of Faraci and Foti (2001), rolling‐grain ripples lasted less than 4 min before developing into vortex ripples when *U*_*o*_ = 0.43 m/s. In the *C*_0_ = 10.6% case, however, the rolling‐grain ripple stage was remarkably prolonged at around 290 min (Figure 2). The similarity between *η* and *d*_*c*_ in the sediment cores indicates that there was insufficient clean sand available beneath the active layer for ripple growth at *t* = 290 min (Figure 5i). However, subsequently as *d*_*c*_ became larger, the ripples continued to grow and reached equilibrium as the clay at the base of the active layer dropped below 8%. A clay content of 8% may therefore be a threshold below which growth toward equilibrium clean‐sand ripples is able to occur, and ripples develop separation vortices typical of vortex ripples. Bed clay contents above 8% thus prevent the circulation cells from supplying enough sand from the troughs to allow the ripples to grow and the flow to separate, so the rolling‐grain ripples persist. This threshold was never passed in the 12.3% clay run, since the clay content below the base of the ripples remained at 10% or above consistently (Figures 5r and 5s), resulting in the persistence of rolling‐grain ripples until the end of the experiment. Further research designed to quantify the clay‐content threshold for the change from rolling‐grain to vortex ripples under different forcing conditions is required to fully understand the influence of cohesive clay on ripple evolution and equilibrium ripple size and shape. This 8% threshold is consistent with Wu et al.’s (2018) experiments, since *C*_0_ ≤ 7.4% for all of their experiments and no reduction in the wave ripple dimensions was found. Also interestingly, Baas et al. (2013) found a drastic reduction in the size of current ripples in runs with *C*_0_ > 13%, with heights and wavelengths lower than 5.5 and 80 mm. These smaller ripples were two‐dimensional and flatter than the three‐dimensional, linguoid, equilibrium clean‐sand ripples (Baas et al., 2013). Most significantly, once formed these small current ripples were stable until the end of the experiments. The postexperiment clay content beneath the active layer was not measured by Baas et al. (2013), as visual observations suggested that the bed had remained relatively unchanged. Within the active layer, the bulk postexperiment clay content was measured, *C*_*r*_, and found to be much reduced by efficient winnowing (0 ≤ *C*_*r*_ ≤ 18%*C*_0_). Thus it is likely that the clay content immediately below the active layer was between *C*_*r*_ and *C*_0_ (as in Figure 5). This would give a representative concentration of ½(*C*_*r*_ + *C*_0_) below the active layer or <7.7% for *C*_0_ ≤ 13% and >7.7% for *C*_0_ > 13%, which is very similar to the 8% threshold.

**Figure 7:**
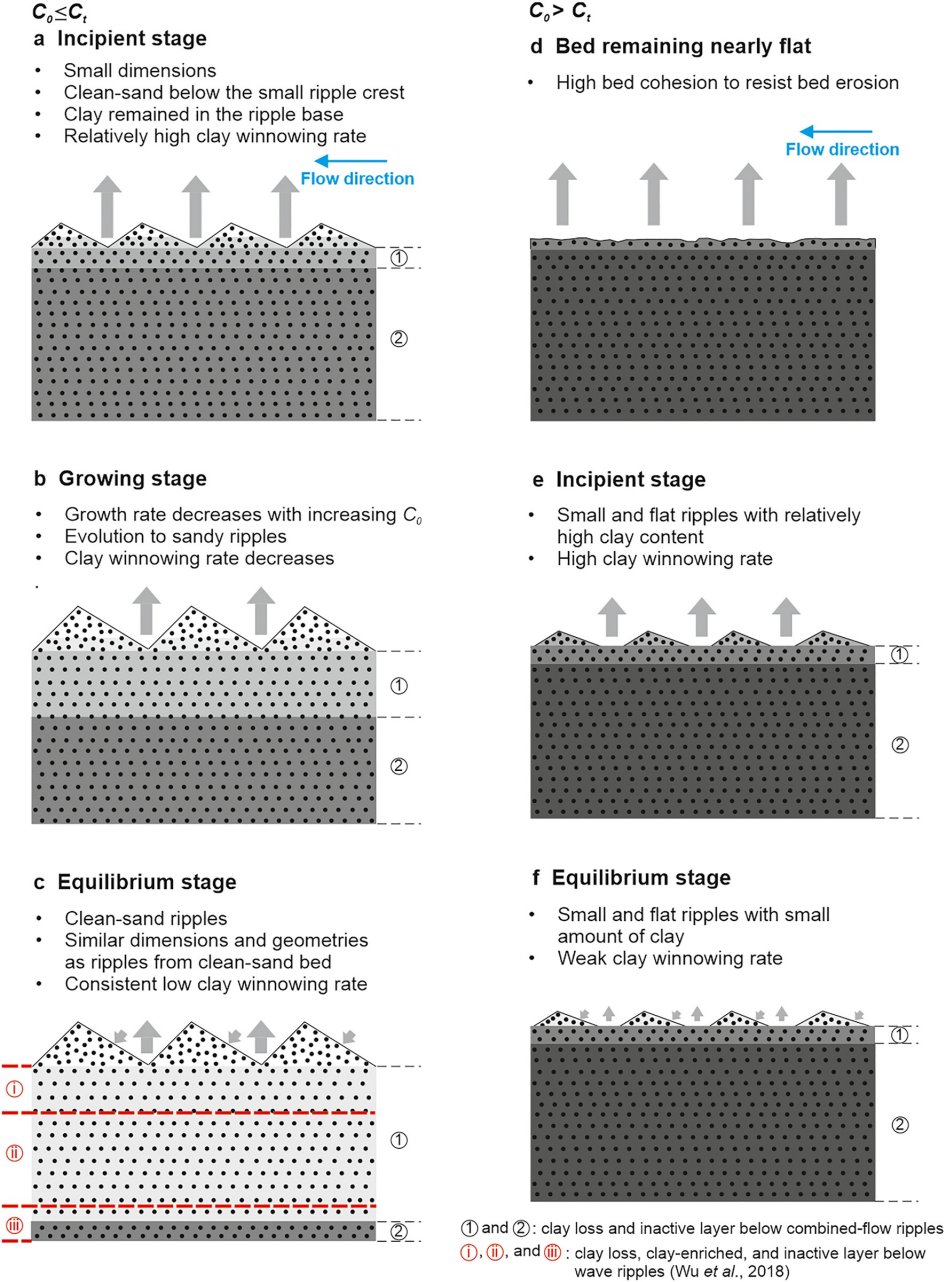
Conceptual models showing the development stages of (a–c) large and (d–f) small equilibrium ripples under currents, waves, and combined flows. *C*_*t*_ is threshold bed clay content. The horizons with different clay fractions are presented by different shades of gray. The gray arrows pointing upward represent clay winnowing and those pointing to the ripple stoss side represent clay entering into bed via hyporheic flow. Red dash lines represent boundaries of sediment layers below the wave ripples of Wu et al. (2018).

It is therefore concluded that two distinct types of equilibrium wave, current, and combined‐flow ripples are able to develop on mixed sand‐clay beds, with the conceptual models of these two types of ripple development shown in Figure 7. If *C*_0_ ≤ *C*_*t*_, the threshold bed clay concentration, relatively large equilibrium ripples, with dimensions and geometries comparable to clean‐sand counterparts, are developed. These ripples experience similar development stages as those of clean‐sand ripples, including incipient, growing, and equilibrium stages (Perillo, Best, Yokokawa, et al., 2014; Figures 7a–7c), although the growth rate is lower than that of clean‐sand ripples.

Relatively small and flat equilibrium ripples are generated as *C*_0_ > *C*_*t*_ (Figures 7d–7f). Incipient ripples that appear after an extended period of flatbed conditions because of high bed cohesion are unable to grow to large sizes because of a lack of clean sand available from beneath the active layer (Figures 7e and 7f). The value of *C*_*t*_ can either be specified for the initial well mixed clay content, *C*_0_, in which case *C*_*t*_ = 10.6% or the clay content at the base of the active layer in which case *C*_*t*_ = 8%. Whereas the threshold for an initial well‐mixed bed may be specific to the wave‐current conditions, the 8% threshold at the base of the active layer appears to be more general.

### 4.2 Deep Cleaning of Clay in Rippled Beds

Our sediment core data confirm earlier findings that winnowing of fine, cohesive material, that is, clay and EPS, from the active layer leads to the transformation of a cohesive bed to sandy ripples (Baas et al., 2013; Malarkey et al., 2015; Wu et al., 2018). Furthermore, the present experimental results demonstrate that clay winnowing rates decrease as the ripples develop (Figures 5 and 7). In the incipient stage of ripple development, the clay in the active layer and immediately below it are easily winnowed as the ripples migrate, resulting in high clay winnowing rates (Figure 7a). As the ripples grow and clay in the active layer is exhausted, such that clay loss only occurs from below the ripple trough, the winnowing efficiency is significantly reduced (Figure 7c). For high clay content cases, *C* > 8%, we infer that the weaker clay winnowing rate, after losing most of the clay contained in the active layer, is limited by stronger cohesion, beneath the ripples (Figure 7f). Importantly, this has the effect of starving the ripples of clean sand so that they can grow no further.

Wu et al. (2018) identified layers enriched in clay relative to initial bed clay content below equilibrium wave ripples (Figure 7d). In the present experiments, clay did accumulate between 25 and 35 mm in the sandy section downstream of the mixed sand‐clay beds (Figures 5f and 5m). This provides further evidence that suspended clay can be carried into rippled beds by hyporheic flow, driven by pressure gradients between ripple troughs and crests (e.g., Dallmann et al., 2020; Huettel et al., 1996; Karwan & Saiers, 2012). Clay probably also entered the rippled beds in the mixed sand‐clay test section, as evidenced by the plateaus of increased clay content immediately below the ripple bases, for example, between 22.5 and 45 mm and between 35 and 55 mm at the end of the 5.7% and 10.6% runs, respectively (Figures 5e and 5l). These depths are similar to the depths at which clay accumulated in the downstream sandy section and in the wave‐ripple experiments of Wu et al. (2018; their Figure 11) but there is still net clay loss at these depths in the mixed sand‐clay test section. We infer that there is a dynamic balance between clay loss and gain below the base of the ripples. Clay gain by downward movement was higher than clay loss by upward winnowing below the base of the wave ripples, causing net clay accumulation at this depth, whereas winnowing‐induced clay loss was dominant over hyporheic clay gain in the present combined‐flow experiments, thus causing “deep cleaning” of bed clay (Figures 7a–7c). The fact that a Gaussian‐type function describes the clay concentration below the active layer implies that the deep cleaning effect is diffusive in nature (e.g., Figure 5e and 5l). Winnowing was stronger under the combined‐flow forcing herein than under the pure wave forcing of Wu et al. (2018), as illustrated by the active‐layer winnowing transport rate estimate being an order of magnitude larger than the hyporheic transport rate estimate in the present experiments. Nevertheless, it is likely that eventually a balance is achieved between active layer winnowing and hyporheic processes as clay is used up in the active layer as ripples develop (*C*_*r*_ → 0), supported by the fact that *T*_*b*_ levels off over time and in the 12.3% case changes sign (Figure 6c).

It is well known that superimposed waves and currents generate high turbulence intensities and shear stresses in the wave boundary layer that are much greater than the sum of their constituents (Grant & Madsen, 1979, Mathisen & Madsen, 1996). Indeed, based on Malarkey and Davies' (2012) method, the maximum skin friction shear stress in the present experiments was 1.41 Pa, c. 40% higher than under wave‐alone conditions with similar wave velocity amplitudes of Wu et al. (2018). This enhanced maximum shear stress, combined with the background turbulence associated with the current maintaining clay in suspension and far larger migration rates (5 ≤ *m* ≤ 20 mm/min), results in much stronger winnowing for wave‐current conditions than wave‐alone conditions. The stress in current‐alone experiments of Baas et al.’s (2013) was the same as the wave‐current mean stress (0.14 Pa) applied here but the ripple migration rates were smaller (1 ≤ *m* ≤ 5 mm/min vs. 5 ≤ *m* ≤ 20 mm/min). Wu (2017) measured ripple migration rates under wave‐alone conditions that were smaller than for the current ripples of Baas et al. (2013), that is, 1 ≤ *m* ≤ 2 mm/min. The rate of winnowing of clay from the current ripples in Baas et al.’s (2013) experiments may therefore have been intermediate between those of Wu (2017) and the present study. Packman and Brooks (2001) quantified the relative importance of winnowing (turnover) to hyporheic pumping using the quantity *U*_p_* = *pm*/*u*_*p*_, where *p* is the porosity (=0.4) and *u*_*p*_ is the pore water velocity (see Supporting Information S1). Winnowing dominates when *U*_p_* >> 1 and hyporheic pumping dominates when *U*_p_* << 1. For the present experiments, *u*_*p*_ = 1.3 mm/min and 1.5 ≤ *U*_p_* ≤ 6.2; for Baas et al. (2013), *u*_*p*_ = 0.2 mm/min and 2 ≤ *U*_p_* ≤ 10; and for Wu et al. (2018), *u*_*p*_ = 0.7 mm/min and 0.6 ≤ *U*_p_* ≤ 1.1. Thus according to this parameter, hyporheic processes are least important for the current‐alone experiments of Baas et al. (2013) and the present experiments and most important for the wave‐alone experiments of Wu et al. (2018). However, even in the wave‐alone experiments winnowing still dominated in the active layer. Only the present wave‐current experiments show the deep cleaning winnowing effect when winnowing is dominant. This is analogous to the “wave‐pumping” effect that produces a deeper hyporheic exchange for wave‐current conditions than wave‐alone conditions when the flow is hyporheically dominated (Clark et al., 2019).

### 4.3 Implications for Natural Environments

The present experiments, supported by Baas et al. (2013) and Wu et al. (2018), show that ripple types change into one another across a narrow range of bed clay contents, suggesting a discontinuity in ripple dimensions and geometries that is not incorporated in mathematical predictors for bedform height and wavelength (e.g., Nelson et al., 2013; Tanaka & Dang, 2006). Because the large equilibrium ripples resemble clean‐sand ripples as a result of highly effective clay winnowing, the application of these predictors may be extended from pure sand beds to weakly cohesive mixed sand‐clay beds. However, these predictors need to be modified to capture the small equilibrium ripples that are stable only on strongly cohesive beds. This indicates that bedform predictors developed from clean‐sand ripples are likely to overpredict ripple roughness for sand beds with a high bed clay content. Indeed, using *k*_*s*_ = 27.7*η* ^2^/*λ* (Li & Amos, 1998), where *k*_*s*_ is the bed roughness due to form drag, the sudden reduction in ripple dimensions at *C*_0_ > 10.6% (Figures 4a and 4b) causes the bed roughness to decrease by an order of magnitude. Brakenhoff et al. (2020) highlighted the fact that small changes in predicted form roughness could result in large changes in sediment transport rate predictions. The data in this study show that such errors may result from neglecting the profound effect of cohesive forces in mixed sand‐clay beds, thus limiting the ability of models to accurately predict changes in the bed morphology of estuaries and coastal seas. The discontinuity between large and small combined‐flow ripples was at an initial bed clay content of 10.6% and at a clay content of c. 8% below the base of the ripples. While it is expected that the 10.6% threshold, which relates to a well‐mixed clay bed, is likely to be dependent on the maximum combined shear stress involved, the 8% condition appears to be more general as it concerns the dynamic nature of cohesive properties in the bed below the ripples. However, both thresholds will be affected by the additional presence of EPS‐induced biological cohesion in sediment in the field (Baas et al., 2019), which has a stronger capacity to resist erosion compared to physical cohesion; small proportions of EPS, of the order of 0.1%, are highly effective in hindering bedform evolution (Malarkey et al., 2015; Parsons et al., 2016). Indeed, Baas et al. (2021) observed a reduction in current ripple height on an intertidal flat during neap tides from c. 20 to c. 10 mm for relatively low clay content between 2% and 5% combined with EPS content between 0.05% and 0.08%. Nonetheless, the findings in this paper should act as a stimulus for further studies toward fully quantifying changes in ripple morphology as a function of hydrodynamic forcing and bed cohesion. This would also be helpful for designing the next‐generation phase diagrams for combined‐flow bedforms, which at present do not cover bed cohesion and therefore cannot predict the small equilibrium ripples found in this study (Dumas et al., 2005; Perillo, Best, & Garcia, 2014).

Our wave‐current experiments show that an apparently stable cohesive mixed sand‐clay bed can become unstable quickly through highly efficient clay winnowing (deep cleaning) far below the bed surface and the active layer, *d*_*c*_ >> *η* (e.g., Figures 5e and 5l). This rapid change from a cohesive substrate to a mobile, predominantly, sandy substrate is likely to occur on intertidal flats under storm‐induced wave‐current flows, as at the beginning of the Dee field campaign (Baas et al., 2021; Lichtman et al., 2018). The rapid change in bed stability may further challenge the modeling of sediment transport in estuaries, given that river flooding often goes hand in hand with storm events (e.g., Gong et al., 2007; Ralston et al., 2013). Such combined flows would not only lead to clay loss by winnowing under high maximum bed shear stress but it may also lead to an increased supply into the estuary of suspended terrestrial clay as well as organic matter and toxic chemicals attracted to clay particle surfaces through physicochemical forces (Partheniades, 2009). These materials could become trapped in the estuary, especially during extended periods of calm conditions, given the slow deposit entry rates discussed by Dallmann et al. (2020). During subsequent storms, however, the strong winnowing‐induced, “deep cleaning” effect is likely to release large volumes of clay, nutrients, and pollutants back into the water column over a short amount of time, with potential impacts on the ecological balance of estuarine and coastal environments and anthropogenic activities. Thus, the findings of this study may stimulate further research into the release of contaminants during storm events and be beneficial for the development of evidence‐based water‐quality regulations in estuarine and coastal environments.

## 5 Conclusions

The present experiments examined the importance of physical cohesion on the size and morphology of ripples generated by combined waves and currents. The experimental data illustrate that with initial clay content, *C*_0_, increasing from 0% to 12.3%, ripple height and wavelength development rates, *r*_*η*_ and *r*_*λ*_, decreased by one order of magnitude from 0.16 to 0.017 mm/min and from 0.25 to 0.052 mm/min, respectively. Clay transport rates out of the bed, as determined by sediment cores measured during the experiment, decreased during ripple development. The experimental results also revealed the development of two distinct types of equilibrium ripples on mixed sand‐clay beds. For *C*_0_ ≤ 10.6%, large two‐dimensional, quasi asymmetric equilibrium ripples developed, with equilibrium height and wavelength, *η*_*e*_ = 14.4 mm; *λ*_*e*_ = 123.9 mm; ripple symmetry index, RSI = 1.4; and ripple steepness, RS ≈ 0.12. These geometric values are close to those of clean‐sand ripples because the winnowing of clay from the developing ripples at these low *C*_0_‐values was highly effective, typically resulting in 100% clay loss in the active layer. Relatively large clay transport rates out of the bed even after equilibrium resulted in clay winnowing extending far below the active layer, as demonstrated by the equivalent clean‐sand depth being far larger than the ripple height. This “deep cleaning” of clay is probably attributable to higher bed shear stresses and migration rates under combined flow than under pure currents and pure waves. In contrast, high bed cohesion with *C*_0_ > 10.6% led to a discontinuity in equilibrium ripple height, generating small, flat, and more asymmetric equilibrium ripples, with *η*_*e*_ and RS collapsing to 4 mm and 0.04, respectively, but RSI increasing to 1.5. This bed‐cohesion discontinuity is compounded by relatively small clay transport rates out of the bed, preventing the erosion of ripple troughs, and therefore limiting the sand supply needed for the growth toward larger clean‐sand ripples. The 10.6% threshold in initial concentration coincides with an 8% concentration threshold at the base of the active layer that inhibits ripple development and is common to other experiments. The experimental findings reemphasize the importance of including clay content in the bedform prediction of sediment transport models for muddy environments, such as estuaries. Moreover, combined wave‐current conditions during storm events have the capacity to winnow large amounts of fine cohesive sediments from the bed, leading to bed instability and water pollution, which may have an impact on existing estuarine environmental regulations.

## Conflict of Interest

The authors declare no conflicts of interest relevant to this study.

## Supporting information

Supporting Information S1

Figure S1

## Data Availability

Supporting data are available through figshare which is a free and open repository (Wu et al., 2021).
