# Supplementary material for: Discontinuity in Equilibrium Wave‐Current Ripple Size and Shape and Deep Cleaning Associated With Cohesive Sand‐Clay Beds
Source: J Geophys Res Earth Surf. 2022 Sep 23;127(9):e2022JF006771. doi: 10.1029/2022JF006771 (PMC9786932; doi:10.1029/2022JF006771)
Supplement: Supplementary file 1 — Supporting Information S1 [file JGRF-127-e2022JF006771-s002.docx]

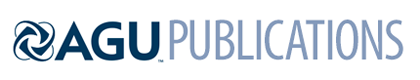


*Journal of Geophysical Research: Earth Surface*

Supporting Information for

**Discontinuity in Equilibrium Wave–Current Ripple Size and Shape and Deep cleaning associated with Cohesive Sand–Clay Beds**

X.Wu^1^, R. Fernández^1^, J. H. Baas^2^, J. Malarkey^1,2^, and D. R. Parsons^1^

^1^Energy and Environment Institute, University of Hull, Hull, UK.

^2^School of Ocean Sciences, Bangor University, Menai Bridge, LL59 5AB, Wales, U.K.

**Contents of this file**

Text S1

**Introduction**

Text S1 describes the method of Packman et al. (2000) to calculate pore water velocity, which is used in Section 4.2 for estimating hyporheic pumping under different flow conditions.

Text S1.

Pore water velocity calculation

The pore water velocity, *u_p_*, which scales the hyporheic processes in the bed, can be calculated by the method of Packman et al. (2000)

| *u_p_* = *kKh_m_* | (S1) |
| --- | --- |

where *k* = 2π/λ, *K* = 600*D*_10_^2^ is the hydraulic conductivity in mm/min, *D*_10_ is the 10-percentile of the grain-size distribution in mm, *h_m_* = 0.14(*U* ^2^/*g*)(*η*/0.34*h*)^3/8^ is the half-amplitude dynamic head, and *U* is the root-mean square flow velocity (Precht and Huettel, 2003). For the present experiments, *U* = 0.28 m/s, *D*_10_ = 0.3 mm and *u_p_* = 1.3 mm/min, for Baas et al. (2013), *U* = 0.4 m/s, *D*_10_ = 0.072 mm and *u_p_* = 0.2 mm/min and for Wu et al. (2018), *U* = 0.19 m/s, *D*_10_ = 0.34 mm and *u_p_* = 0.7 mm/min.
