# Supplementary material for: Discontinuity in Equilibrium Wave‐Current Ripple Size and Shape and Deep Cleaning Associated With Cohesive Sand‐Clay Beds
Source: J Geophys Res Earth Surf. 2022 Sep 23;127(9):e2022JF006771. doi: 10.1029/2022JF006771 (PMC9786932; doi:10.1029/2022JF006771)
Supplement: Supplementary file 2 — Figure S1 [file JGRF-127-e2022JF006771-s001.docx]

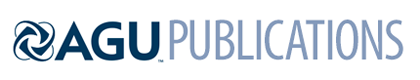


*Journal of Geophysical Research: Earth Surface*

Supporting Information for

**Discontinuity in Equilibrium Wave–Current Ripple Size and Shape and Deep cleaning associated with Cohesive Sand–Clay Beds**

X.Wu^1^, R. Fernández^1^, J. H. Baas^2^, J. Malarkey^1,2^, and D. R. Parsons^1^

^1^Energy and Environment Institute, University of Hull, Hull, UK.

^2^School of Ocean Sciences, Bangor University, Menai Bridge, LL59 5AB, Wales, U.K.

**Contents of this file**

Figure S1

**Introduction**

Figure S1 shows consistent vertical bed clay content before experiment start.

**
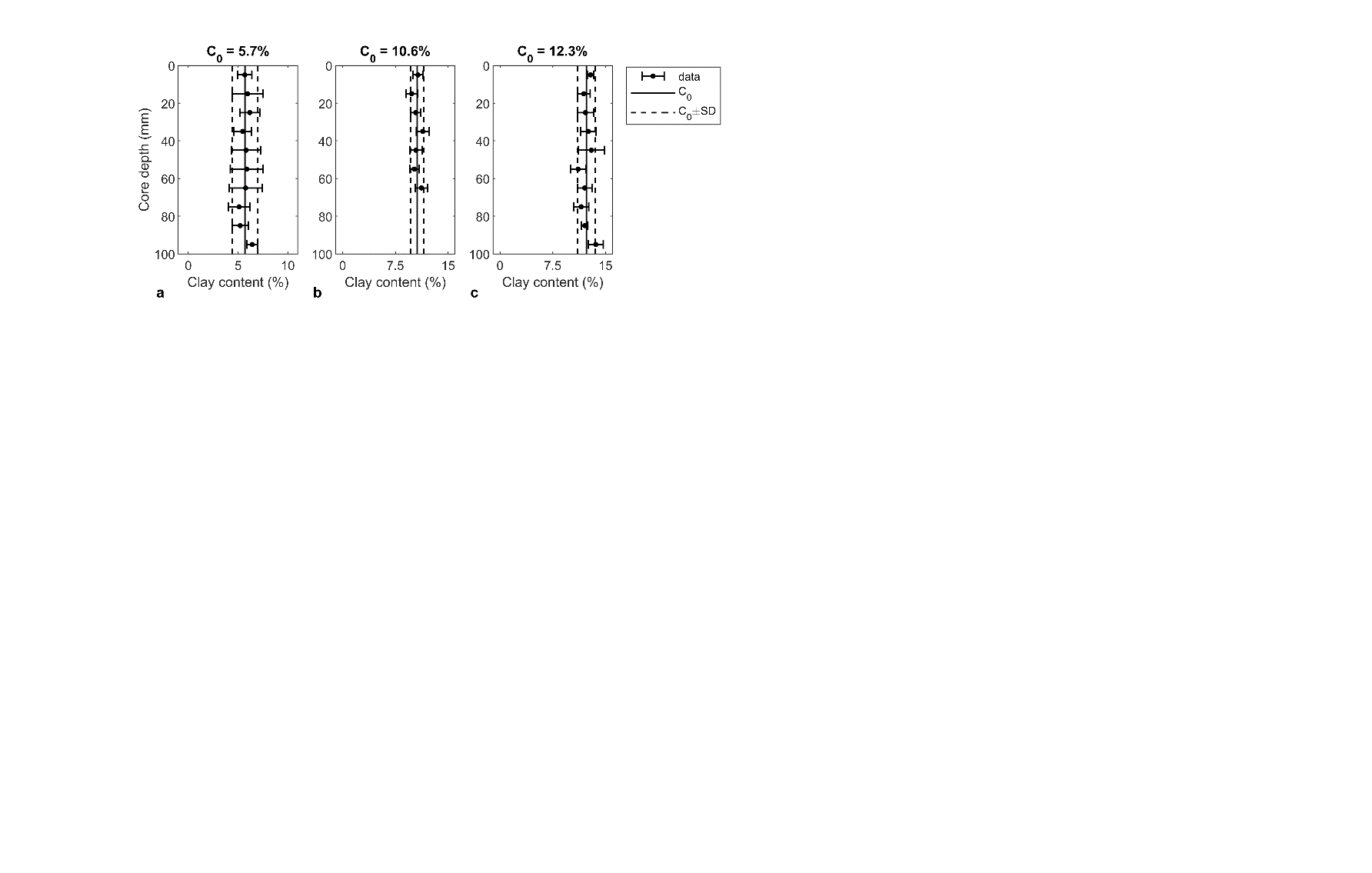
**

Figure S1. Initial clay content in the three experimental cases considered in Figure. 5 (*C_0_* = 5.7, 10.6 and 12.3%) showing the data and one standard deviation at each level and the mean and standard deviation over all depths.
